# Negative Association between Serum Parathyroid Hormone Levels and Urinary Perchlorate, Nitrate, and Thiocyanate Concentrations in U.S. Adults: The National Health and Nutrition Examination Survey 2005–2006

**DOI:** 10.1371/journal.pone.0115245

**Published:** 2014-12-16

**Authors:** Wen-Ching Ko, Chien-Liang Liu, Jie-Jen Lee, Tsang-Pai Liu, Po-Sheng Yang, Yi-Chiung Hsu, Shih-Ping Cheng

**Affiliations:** 1 Department of Surgery, MacKay Memorial Hospital and Mackay Medical College, Taipei, Taiwan; 2 Mackay Junior College of Medicine, Nursing, and Management, Taipei, Taiwan; 3 Department of Pharmacology and Graduate Institute of Medical Sciences, Taipei Medical University, Taipei, Taiwan; 4 Institute of Statistical Science, Academia Sinica, Taipei, Taiwan; Ecole Normale Supérieure de Lyon, France

## Abstract

**Objectives:**

Perchlorate, nitrate, and thiocyanate are well-known inhibitors of the sodium-iodide symporter and may disrupt thyroid function. This exploratory study investigated the association among urinary perchlorate, nitrate, and thiocyanate concentrations and parathyroid hormone (PTH) levels in the general U.S. population.

**Methods:**

We analyzed data on 4265 adults (aged 20 years and older) from the National Health and Nutrition Examination Survey in 2005 through 2006 to evaluate the relationship among urinary perchlorate, nitrate, and thiocyanate concentration and PTH levels and the presence of hyperparathyroidism cross-sectionally.

**Results:**

The geometric means and 95% confidence interval (95% CI) concentrations of urinary perchlorate, nitrate, and thiocyanate were 3.38 (3.15–3.62), 40363 (37512–43431), and 1129 (1029–1239) ng/mL, respectively. After adjusting for confounding variables and sample weights, creatinine-corrected urinary perchlorate was negatively associated with serum PTH levels in women (P = 0.001), and creatinine-corrected urinary nitrate and thiocyanate were negatively associated with serum PTH levels in both sex groups (P = 0.001 and P<0.001 for men, P = 0.018 and P<0.001 for women, respectively). Similar results were obtained from sensitivity analyses performed for exposure variables unadjusted for creatinine with urinary creatinine added as a separate covariate. There was a negative relationship between hyperparathyroidism and urinary nitrate and thiocyanate [odds ratio (95% CI) = 0.77 (0.60–0.98) and 0.69 (0.61–0.79), respectively].

**Conclusions:**

A higher urinary concentration of perchlorate, nitrate, and thiocyanate is associated with lower serum PTH levels. Future studies are needed to determine the pathophysiological background of the observation.

## Introduction

Monovalent anions such as perchlorate, nitrate, fluoroborate, and thiocyanate are known to competitively inhibit iodide uptake and may disrupt thyroid function [1], [2]. Our knowledge of the human health effects of perchlorate mainly comes from the use of potassium perchlorate in the treatment of hyperthyroidism [3]. Used as an oxidizer in propellants and rocket fuels, perchlorate is highly water-soluble and has been detected in drinking water and natural waters [4]. For U.S. adults, vegetables and dairy products are major contributor of perchlorate in the diet [5]. Perchlorate taken into the body is rapidly eliminated in the urine, and measurement of urinary perchlorate is helpful to assess recent exposure.

Nitrate is the final breakdown product of nitrogen fertilizers. The majority of nitrate intake comes from drinking water and food [6]. A study showed that higher nitrate levels in public water supplies were associated with an increased risk of thyroid cancer, and higher intake of dietary nitrate was associated with an increased risk of thyroid cancer and hypothyroidism [7]. Thiocyanate enters the body from the diet (such as cruciferous vegetables) or is synthesized from cyanide by sulfur transferase enzymes. Smoking cigarettes is the major sources of cyanide exposure for those who do not work in cyanide-related industries. Thiocyanate level can be used as an indicator for tobacco smoke exposure, but there is a large overlap between smokers and nonsmokers because of numerous other sources for cyanide [8]. In general, risk assessment for perchlorate exposure should consider co-exposure to nitrate and thiocyanate [9].

Parathyroid hormone (PTH) has a principal biological function in maintaining calcium and phosphate homeostasis. The secretion of PTH is mainly regulated by the amount of circulating ionized calcium via the calcium-sensing receptor (CaSR) located on the surface of the chief cells [10]. In addition, extracellular calcium stimulates vitamin D receptor (VDR) expression in parathyroid glands [11]. Although 1,25(OH)_2_ vitamin D decreases PTH gene transcription through VDR, studies in VDR knock-out mice suggest that vitamin D pathways play a secondary role in parathyroid hyperplasia [12]. Hyperparathyroidism is defined by an increased activity of the parathyroid glands, either from an intrinsic abnormal change altering PTH excretion (primary or tertiary) or from an extrinsic change stimulating PTH production (secondary) [13]. Primary hyperparathyroidism is the third most common endocrine disorder. Clinical presentations have remarkably changed since the development of automated serum calcium measurement in the early 1970s. Recent epidemiological data suggest that primary hyperparathyroidism is increasingly prevalent [14]. The effects of environmental, nutritional, and iatrogenic factors are poorly defined.

Using the National Health and Nutrition Examination Survey (NHANES) data, Paik and colleagues demonstrated that smokers and males had lower PTH levels [15]. Furthermore, serum PTH levels were independently associated with blood pressure and with the presence of hypertension or prehypertension among U.S. adults [16]. To date there is no study specifically investigating the relationship between PTH levels and the effects of various monovalent anions that have traditionally been considered as thyroid-disrupting agents. The aim of this exploratory study is to evaluate the association between urinary concentrations of perchlorate, nitrate, and thiocyanate and serum PTH level as well as the presence of hyperparathyroidism in U.S. adults. The results from this study may provide some insights into to non-classical regulatory mechanisms of PTH secretion and potential contributing factors to hyperparathyroidism.

## Methods

### Study Design and Population

Data were obtained from the 2005–2006 NHANES. NHANES is a nationally representative cross-sectional survey designed to collect information on the health and nutrition status of the U.S. civilian noninstitutionalized population. Survey data are published biannually. A detailed description of sampling and data collection procedures is available on the Centers for Disease Control and Prevention website [17]. Interviews were conducted with all participants by trained personnel using standardized procedures. Information on age, race/ethnicity, and cigarette smoking was collected during the interview. Ethical approval for the study was obtained from the Research Ethics Review Board of National Center for Health Statistics. Written informed consent was given by all participants.

### Laboratory Assessment

Serum total calcium was measured by an indirect ion-selective electrode method (Beckman LX20). When serum albumin level was <4.0 g/dL, calcium levels were corrected using the following formula: corrected calcium (mg/dL) = measured total calcium (mg/dL) +0.8 * [4.0 - serum albumin (g/dL)]. Serum 25-hydroxyvitamin D level was determined using a Diasorin (formerly Incstar) 25(OH)D assay. Serum PTH level was measured by electrochemiluminescence immunoassay on an Elecsys 1010 autoanalyzer (Roche Diagnostics). Urine samples were analyzed for perchlorate, nitrate, and thiocyanate in participants aged 6 years and older. Nonetheless, the analysis of this study was limited to participants 20 years of age and older. The quantitative measurement of perchlorate, nitrate, and thiocyanate in human urine was performed using ion chromatography coupled with electrospray tandem mass spectrometry. The lower detection limits were 0.05 ng/mL for urinary perchlorate, 700 ng/mL for urinary nitrate, and 20 ng/mL for urinary thiocyanate. For concentrations less than the limit of detection, a value equal to the detection limit divided by the square root of two was used.

### Statistical Analysis

All statistical analyses were computed by using survey commands of STATA (STATA Corporation) to incorporate sample weights and to adjust for clusters and strata of the complex sample design. Our study focused on the 2,387 men and 2,592 women 20 years and older who participated in NHANES 2005–2006. Subjects with missing PTH (n = 502), urinary perchlorate, nitrate, or thiocyanate data (n = 442) were excluded. We also excluded participants with missing data for smoking status (n = 2), body mass index (n = 47), calcium level (n = 12), and 25-hydroxyvitamin D level (n = 1). A total of 4,265 NHANES participants were included in the final analyses.

Respondents who had smoked at least 100 cigarettes during their lifetime and, at the time of interview, reported smoking every day or some days were classified as current smokers. Respondents who had smoked fewer than 100 cigarettes in their lifetime were classified as never smokers. Concentrations of urinary perchlorate, nitrate, and thiocyanate are expressed as the geometric mean with a 95% confidence interval in different subgroups and were tested by linear regression to assess independent demographic predictors of urinary measurements.

Due to significant deviation from the normal distribution, the natural log transformation was performed for PTH and urinary measurements. Urinary measurements were normalized for creatinine as follows: urinary anion concentration (ng/mL)/urinary creatinine (mg/dL)/100 = µg anion/g creatinine. We constructed full multivariable linear regression models with serum PTH levels as the dependent variable and individual natural log-transformed creatinine-corrected urinary measurements as a predictor along with age (continuous variable), race and ethnicity (categorical), smoking status (categorical), and body mass index (continuous) as covariates. Corrected total calcium and 25-hydroxyvitamin D levels, both being important determinants of serum PTH levels, were included in the final model.

To evaluate dose-response effects across the population, the urinary measurements were also stratified across the population in quartiles. Sample weights, which account for the differential probabilities of selection, nonresponse and noncoverage, were incorporated into the variance estimation process to be representative of the US population.

In our analyses, urinary measurements were divided by the creatinine concentration to adjust for dilution. However, urinary creatinine concentration may vary by age, sex, and race/ethnicity. We avoided this limitation by performing analyses in adults because creatinine adjustment elevates the urinary chemical concentrations in children compared with adults [18]. Nonetheless, we have also explored an alternative approach to separate the urinary anion concentration from the urinary creatinine concentration in the regression models.

In logistic regression, we used the same model with sample weights to test urinary measurements related to the odds ratio (OR) of hyperparathyroidism (defined as PTH >70 pg/mL). To evaluate interactions between urinary measurements, the synergism index (SI) was calculated as follows: SI = [OR_11_−1]/([OR_01_+OR_10_] −2), where OR_11_ is equal to OR of the joint effect of two factors and OR_10_ and OR_01_ are equal to OR of each risk factor in the absence of the other [19]. A value greater than unity was indicative of synergism.

## Results

Table 1 shows the basic demographics of the sample population. The study sample consisted of 2,058 men and 2,207 women. Males were found to have significantly higher geometric mean urinary perchlorate, nitrate, and thiocyanate levels when compared with females. Older age was associated with lower levels of urinary nitrate and thiocyanate. Of note, current smokers were found to have significantly higher levels of urinary nitrate and thiocyanate as well as a lower urinary perchlorate level. Urinary nitrate levels were moderately and positively correlated with urinary perchlorate and thiocyanate. Urinary perchlorate and thiocyanate levels were weakly correlated.

**Table 1:** Demographics and geometric means with 95% confidence intervals of urinary concentrations of perchlorate, nitrate, and thiocyanate among the United States adults, NHANES 2005–2006.

	No.	Perchlorate (ng/mL)	Nitrate (ng/mL)	Thiocyanate (ng/mL)
Total	4265	3.38 (3.15–3.62)	40363 (37512–43431)	1129 (1029–1239)
Gender				
Male	2058	3.80 (3.56–4.07)***	44979 (41882–48305)***	1361 (1231–1505)***
Female	2207	3.02 (2.78–3.28)***	36486 (33574–39651)***	948 (858–1048)***
Age (years)				
<40	1627	3.50 (3.28–3.72)	45691 (42202–49467)***	1303 (1148–1478)***
<60	1343	3.28 (2.94–3.67)	42730 (39372–46374)***	1348 (1208–1504)***
≥60	1295	3.33 (3.09–3.58)	32559 (30681–34552)***	785 (718–859)***
Race				
Mexican American	877	3.89 (3.59–4.22)**	42713 (39278–46447)	714 (652–781)***
Non-Hispanic white	2143	3.25 (2.93–3.61)**	38261 (34787–42081)	1247 (1120–1388)***
Non-Hispanic black	948	3.26 (2.92–3.63)**	42494 (38648–46722)	1469 (1286–1677)***
Other	297	3.25 (2.85–3.70)**	42632 (37754–48140)	922 (689–1235)***
Smoking status				
Current smoker	942	3.13 (2.82–3.47)*	48698 (44212–53640)***	4044 (3661–4468)***
Former smoker	1083	3.48 (3.23–3.74)*	36783 (34185–39578)***	819 (737–909)***
Never smoker	2240	3.44 (3.22–3.67)*	39012 (36008–42267)***	771 (701–848)***
Body mass index (kg/m^2^)				
<25	1288	3.08 (2.76–3.44)**	38395 (34726–42452)*	1106 (950–1289)
<30	1465	3.53 (3.22–3.87)**	40898 (38073–43932)*	1062 (974–1158)
≥30	1512	3.49 (3.32–3.67)**	41585 (38328–45119)*	1218 (1117–1330)

*P<0.05, **P<0.01, ***P<0.001.

**Figure 1:**
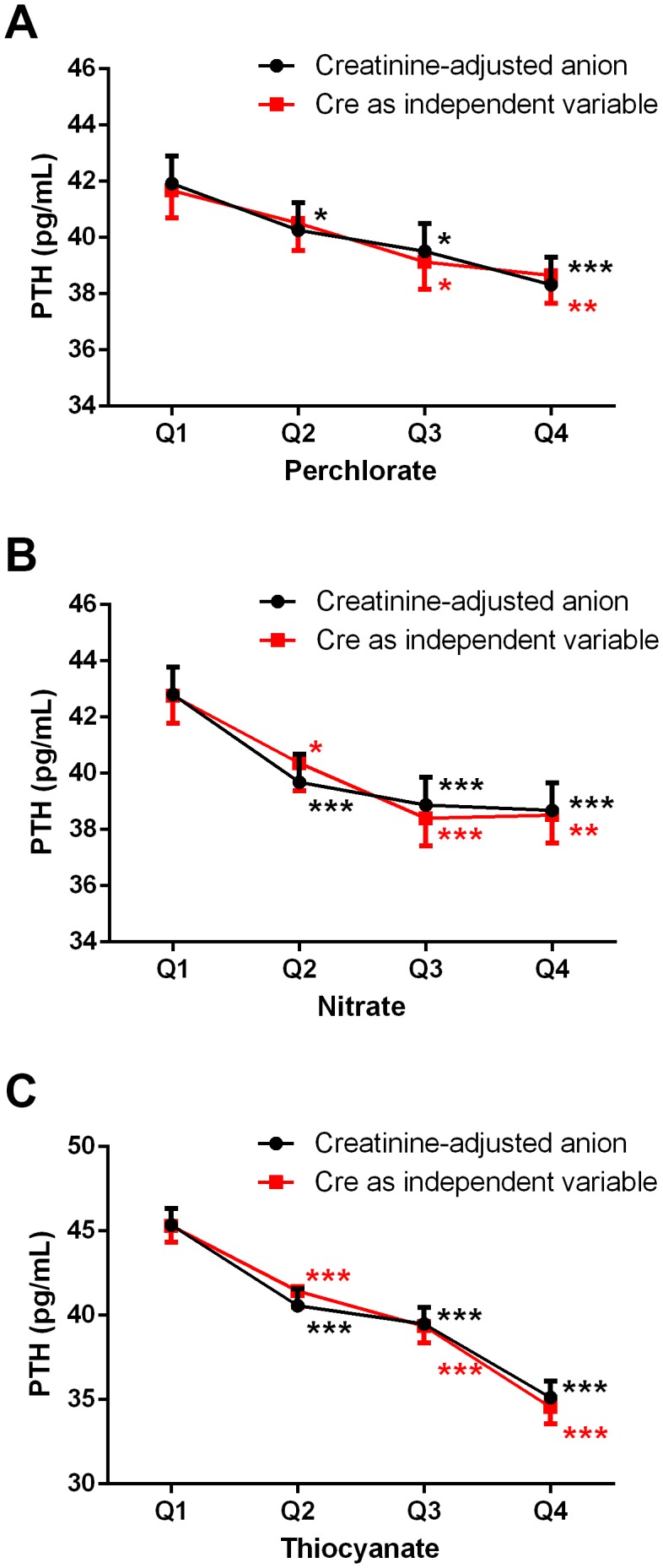
Weighted mean ± SE of serum parathyroid hormone (PTH) levels versus urinary concentrations of perchlorate, nitrate, and thiocyanate among the United States adults, NHANES 2005–2006. Analyses were performed with two approaches: (1) creatinine-corrected urinary measurements, and (2) analyte concentration unadjusted for creatinine but urinary creatinine was included as a separate independent variable. **A**, Serum PTH levels by urinary perchlorate quartiles. **B**, Serum PTH levels by urinary nitrate quartiles. **C**, Serum PTH levels by urinary thiocyanate quartiles. Adjusted for age, race/ethnicity, smoking status, body mass index, corrected total serum calcium, and 25-hydroxyvitamin D levels. *P<0.05, **P<0.01, ***P<0.001 versus first quartile.

Both corrected serum calcium and 25-hydroxyvitamin D concentrations were negatively correlated with urinary nitrate but not perchlorate or thiocyanate levels. Serum calcium and 25-hydroxyvitamin D were included in the final multivariable regression model because they are important determinants of PTH production and secretion. The results of multivariable linear regression analyses are shown in Table 2. We observed a negative relationship between serum PTH levels and creatinine-corrected urinary perchlorate concentrations in women. There were negative associations between serum PTH levels and urinary nitrate and thiocyanate in both men and women. To evaluate dose-response effects across the population, the creatinine-corrected urinary measurements were stratified across the population in quartiles (Table 3). The mean serum PTH levels decreased significantly with increasing quartiles of urinary nitrate and thiocyanate concentrations in men. In women, serum PTH levels significantly decreased as urinary perchlorate, nitrate and thiocyanate concentrations increased from first to fourth quartile. These results were consistent with the results obtained when creatinine-corrected urinary measurements were entered as a continuous variable in linear regression models. A summary of the association among quartiles of creatinine-corrected urinary measurements and serum PTH levels after adjusting for potential covariates in men and women is illustrated in Fig. 1.

**Table 2:** Adjusted regression coefficients with 95% confidence intervals (95% CIs) by multiple linear regression analysis for serum parathyroid hormone levels and creatinine-corrected urinary concentrations of perchlorate, nitrate, and thiocyanate among the United States adults, NHANES 2005–2006.

	Model 1				Model 2			
	Men		Women		Men		Women	
	β (95% CI)	*P* value	β (95% CI)	*P* value	β (95% CI)	*P* value	β (95% CI)	*P* value
Perchlorate	−0.02 (−0.05 to 0.01)	0.124	−0.07 (−0.10 to −0.04)	<0.001	−0.03 (−0.05 to 0.00)	0.084	−0.05 (−0.08 to −0.02)	0.001
Nitrate	−0.06 (−0.09 to −0.03)	0.002	−0.05 (−0.09 to −0.01)	0.022	−0.07 (−0.10 to −0.03)	0.001	−0.05 (−0.08 to −0.01)	0.018
Thiocyanate	−0.09 (−0.11 to −0.07)	<0.001	−0.10 (−0.12 to −0.09)	<0.001	−0.09 (−0.11 to −0.08)	<0.001	−0.10 (−0.11 to −0.08)	<0.001

Model 1: adjusted for age (continuous), race/ethnicity, smoking status, and body mass index (continuous); Model 2: adjusted for variables in Model 1 plus corrected total serum calcium and 25-hydroxyvitamin D levels (both continuous).

**Table 3:** Weighted mean ± SE of serum parathyroid hormone levels (pg/mL) across quartiles of creatinine-corrected urinary concentrations of perchlorate, nitrate, and thiocyanate among the United States adults, NHANES 2005–2006.

	Men			Women		
	Crude	Model 1	Model 2	Crude	Model 1	Model 2
Perchlorate						
≤25^th^	46.7±1.1	42.4±1.0	42.1±1.0	47.7±1.3	42.7±1.0	42.0±1.0
25th to 50th	44.3±1.2	39.7±1.0	39.7±1.0	45.8±1.5	41.2±1.0	41.1±1.0
50th to 75th	45.2±0.9	40.0±1.0	39.9±1.0	43.4±1.1	39.0±1.0	39.7±1.0
>75th	46.5±1.6	41.0±1.0	40.6±1.0	44.2±1.3	37.8±1.0	38.5±1.0
*P* value for trend	0.644	0.292	0.216	0.001	0.001	0.007
Nitrate						
≤25th	51.6±1.0	42.9±1.0	43.0±1.0	52.2±1.7	42.9±1.0	43.0±1.0
25th to 50th	43.7±0.8	39.6±1.0	39.6±1.0	44.9±1.1	39.9±1.0	40.1±1.0
50th to 75th	41.8±0.8	39.5±1.0	39.1±1.0	42.2±1.1	39.0±1.0	39.2±1.0
>75th	44.6±1.2	40.9±1.0	40.3±1.0	43.2±1.0	38.8±1.0	39.1±1.0
*P* value for trend	<0.001	0.040	0.004	<0.001	0.005	0.001
Thiocyanate						
≤25th	52.9±1.2	45.5±1.0	45.1±1.0	53.9±1.3	45.4±1.0	45.5±1.0
25th to 50th	46.5±1.4	41.1±1.0	41.0±1.0	44.1±1.0	40.1±1.0	40.3±1.0
50th to 75th	45.0±1.1	40.2±1.0	40.4±1.0	42.3±0.9	38.5±1.0	39.1±1.0
>75th	39.0±1.1	36.5±1.0	36.1±1.0	41.2±1.4	37.1±1.0	37.1±1.0
*P* value for trend	<0.001	<0.001	<0.001	<0.001	<0.001	<0.001

Model 1: adjusted for age (continuous), race/ethnicity, smoking status, and body mass index (continuous); Model 2: adjusted for variables in Model 1 plus corrected total serum calcium and 25-hydroxyvitamin D levels (both continuous).

Sensitivity analyses were performed for exposure variables (unadjusted for creatinine) with urinary creatinine added as a separate covariate. As shown in Table 4, the results from multivariable linear regression analyses were very similar to those in Table 2. Namely, there was a negative relationship between serum PTH levels and urinary perchlorate in women, whereas there were negative associations between serum PTH levels and urinary nitrate and thiocyanate in both men and women. Likewise, similar results were obtained from analyzing the associations among quartiles of unadjusted urinary measurements and PTH levels (Fig. 1). Taken together, serum PTH levels negatively correlated with urinary perchlorate, nitrate, and thiocyanate, either adjusted or unadjusted for urinary creatinine.

**Table 4:** Adjusted regression coefficients with 95% confidence intervals (95% CIs) by multiple linear regression analysis for serum parathyroid hormone levels and urinary concentrations of perchlorate, nitrate, and thiocyanate (unadjusted for creatinine) among the United States adults, NHANES 2005–2006.

	Model 1				Model 2			
	Men		Women		Men		Women	
	β (95% CI)	*P* value	β (95% CI)	*P* value	β (95% CI)	*P* value	β (95% CI)	*P* value
Perchlorate	−0.02 (−0.05 to 0.01)	0.111	−0.05 (−0.08 to −0.03)	<0.001	−0.03 (−0.06 to 0.00)	0.064	−0.04 (−0.06 to −0.02)	0.001
Nitrate	−0.06 (−0.10 to −0.03)	0.001	−0.03 (−0.07 to 0.01)	0.099	−0.07 (−0.10 to −0.04)	<0.001	−0.04 (−0.07 to 0.00)	0.043
Thiocyanate	−0.10 (−0.12 to −0.08)	<0.001	−0.09 (−0.11 to −0.07)	<0.001	−0.11 (−0.12 to −0.09)	<0.001	−0.09 (−0.11 to −0.08)	<0.001

Model 1: adjusted for age (continuous), race/ethnicity, smoking status, body mass index (continuous), and urinary creatinine (continuous); Model 2: adjusted for variables in Model 1 plus corrected total serum calcium and 25-hydroxyvitamin D levels (both continuous).

**Figure 2:**
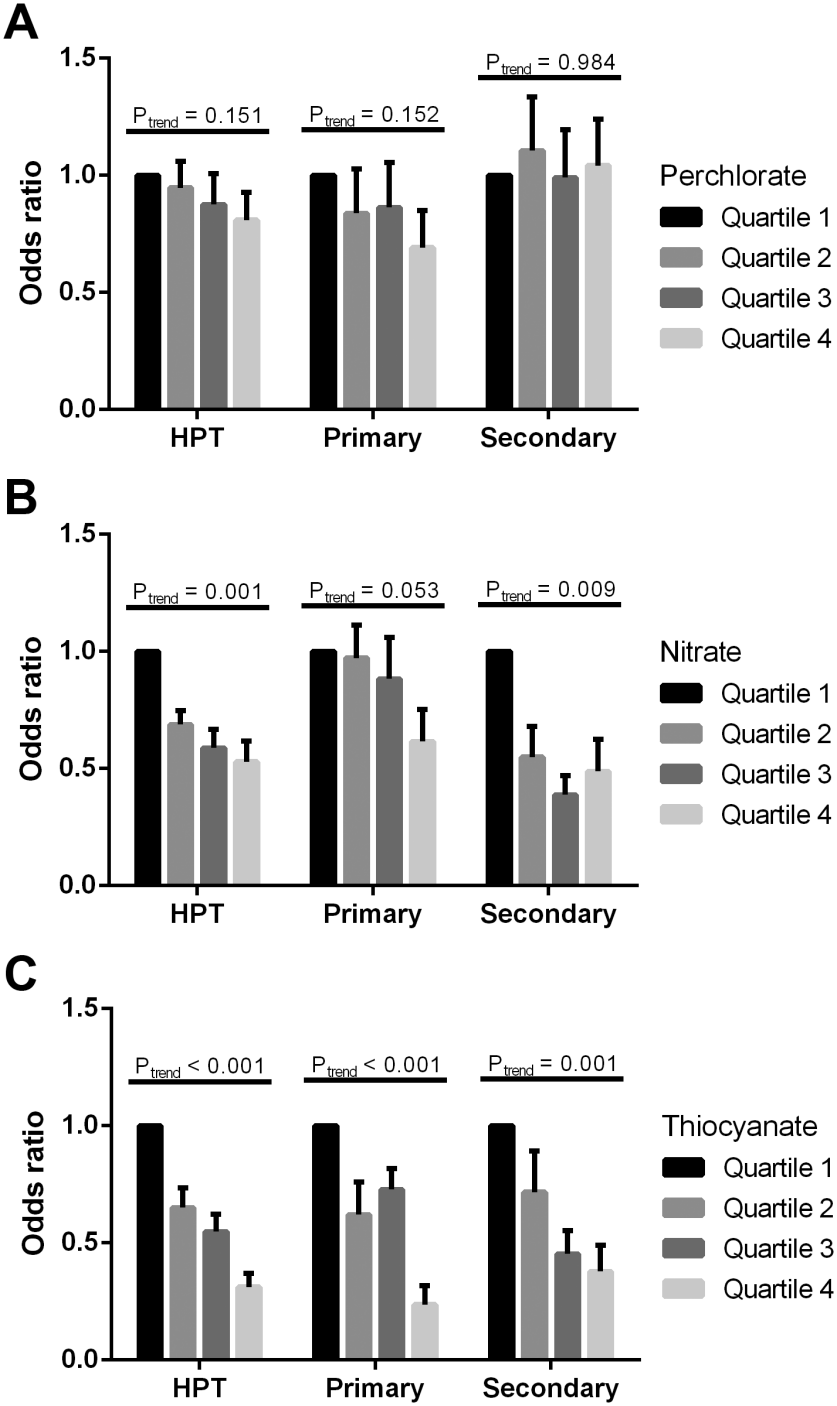
Adjusted odds ratio ± SE for hyperparathyroidism (HPT, defined as parathyroid hormone >70 pg/mL) by creatinine-corrected urinary concentrations of perchlorate, nitrate, and thiocyanate among the United States adults, NHANES 2005–2006. Primary HPT was arbitrarily defined as albumin-corrected total serum calcium ≥9.5 mg/dL, and secondary HPT was defined as corrected calcium <9.5 mg/dL. **A**, Odd ratios by urinary perchlorate quartiles. **B**, Odd ratios by urinary nitrate quartiles. **C**, Odd ratios by urinary thiocyanate quartiles. Adjusted for age, race/ethnicity, smoking status, body mass index, corrected total serum calcium, and 25-hydroxyvitamin D levels.

Among the 4,265 participants who formed our analysis sample, 449 (237 women and 212 men) had hyperparathyroidism (serum PTH >70 pg/mL). In logistic regression models adjusting for age, race/ethnicity, smoking, body mass index, corrected total calcium and 25-hydroxyvitamin D levels, there was no association between natural log-transformed creatinine-corrected perchlorate levels and hyperparathyroidism in both women and men (Table 5). There was a negative association between log-transformed creatinine-corrected urinary nitrate and thiocyanate and hyperparathyroidism in women (OR = 0.75 and 0.66; 95% CI = 0.60–0.94 and 0.59–0.74, respectively) and in men (OR = 0.65 and 0.66; 95% CI = 0.54–0.79 and 0.59–0.74, respectively). Consistently, hyperparathyroidism was negatively associated with increasing quartiles of creatinine-corrected urinary nitrate and thiocyanate levels. These findings are illustrated graphically in Fig. 2.

**Table 5:** Adjusted odds ratios (ORs) with 95% confidence intervals (95% CIs) for hyperparathyroidism (defined as parathyroid hormone>70 pg/mL) by creatinine-corrected urinary concentrations of perchlorate, nitrate, and thiocyanate among the United States adults, NHANES 2005–2006.

	Model 1				Model 2			
	Men		Women		Men		Women	
	OR (95% CI)	*P* value	OR (95% CI)	*P* value	OR (95% CI)	*P* value	OR (95% CI)	*P* value
Perchlorate	0.96 (0.77–1.18)	0.670	0.85 (0.68–1.07)	0.152	0.96 (0.77–1.19)	0.660	0.91 (0.74–1.12)	0.346
Nitrate	0.67 (0.55–0.80)	<0.001	0.73 (0.60–0.89)	0.004	0.65 (0.54–0.79)	<0.001	0.75 (0.60–0.94)	0.015
Thiocyanate	0.66 (0.59–0.74)	<0.001	0.65 (0.58–0.73)	<0.001	0.66 (0.59–0.74)	<0.001	0.66 (0.59–0.74)	<0.001

Model 1: adjusted for age (continuous), race/ethnicity, smoking status, and body mass index (continuous); Model 2: adjusted for variables in Model 1 plus corrected total serum calcium and 25-hydroxyvitamin D levels (both continuous).

We arbitrarily defined primary hyperparathyroidism as albumin-corrected total serum calcium ≥9.5 mg/dL (unsuppressed serum calcium with an elevated PTH level, n = 212), and secondary hyperparathyroidism as calcium <9.5 mg/dL (n = 237). As shown in Table 6, urinary nitrate was negatively associated with primary hyperparathyroidism in men and secondary hyperparathyroidism in both sex cohorts. The negative associations between urinary thiocyanate and primary and secondary hyperparathyroidism were present in both sex groups. Collectively, these findings further corroborate that the association between these anions and PTH levels is independent of calcium concentrations.

**Table 6:** Adjusted odds ratios (ORs) with 95% confidence intervals (95% CIs) for primary hyperparathyroidism (defined as parathyroid hormone >70 pg/mL and corrected calcium ≥9.5 mg/dL) and secondary hyperparathyroidism (defined as parathyroid hormone >70 pg/mL and corrected calcium <9.5 mg/dL) by creatinine-corrected urinary concentrations of perchlorate, nitrate, and thiocyanate among the United States adults, NHANES 2005–2006.

	Primary hyperparathyroidism				Secondary hyperparathyroidism			
	Men		Women		Men		Women	
	OR (95% CI)	*P* value	OR (95% CI)	*P* value	OR (95% CI)	*P* value	OR (95% CI)	*P* value
Perchlorate	0.87 (0.64–1.17)	0.325	0.97 (0.71–1.32)	0.839	1.00 (0.64–1.56)	0.994	0.99 (0.76–1.29)	0.957
Nitrate	0.64 (0.46–0.88)	0.010	0.87 (0.61–1.26)	0.452	0.64 (0.41–0.98)	0.042	0.73 (0.55–0.96)	0.027
Thiocyanate	0.61 (0.45–0.82)	0.003	0.76 (0.65–0.89)	0.002	0.70 (0.56–0.88)	0.005	0.67 (0.53–0.84)	0.002

Note: adjusted for age (continuous), race/ethnicity, smoking status, body mass index (continuous), corrected total serum calcium (continuous), and 25-hydroxyvitamin D levels (continuous).

The SI proposed by Rothman was computed to assess the empirical deviation from the additive interaction relationship [19]. Table 7 shows the synergistic interaction between urinary measurements in influencing hyperparathyroidism. The highest interaction was found between low perchlorate level and low nitrate level. Furthermore, there was an indication for the synergism between low nitrate level and low thiocyanate level. These findings are in correspondence with the positive correlation between urinary nitrate and urinary perchlorate and thiocyanate levels.

**Table 7:** Synergistic interaction between creatinine-corrected urinary concentrations of perchlorate, nitrate, and thiocyanate in influencing hyperparathyroidism (defined as parathyroid hormone >70 pg/mL) among the United States adults, NHANES 2005–2006.

		Men		Women	
Variables		Adjusted OR	Synergistic interaction	Adjusted OR	Synergistic interaction
Perchlorate Q1	Nitrate Q1				
No	No (reference)	1	2.40	1	2.17
No	Yes	1.65		1.37	
Yes	No	0.77		0.86	
Yes	Yes	2.01		1.50	
Nitrate Q1	Thiocyanate Q1				
No	No (reference)	1	1.68	1	1.31
No	Yes	1.33		1.93	
Yes	No	1.62		1.24	
Yes	Yes	2.60		2.53	
Perchlorate Q1	Thiocyanate Q1				
No	No (reference)	1	0.98	1	1.14
No	Yes	1.67		2.02	
Yes	No	1.18		0.92	
Yes	Yes	1.83		2.07	

OR: odds ratio for hyperparathyroidism; Q1: first quartile.

## Discussion

In this large, cross-sectional study of national representative sample of adults in the U.S., we for the first time found a negative association between serum PTH levels and exposure to perchlorate, nitrate, and thiocyanate.

Early in the evolution of life, calcium was plenty available and phosphate supply was limited in seawater. To cope with the challenge of higher gravity and calcium-poor environment, terrestrial animals use bone as calcium reservoir and develop PTH and vitamin D system as the main regulators of calcium homeostasis, and fibroblast growth factor-23 as the dominant factor regulating phosphate homeostasis [20]. Minute-to-minute changes in PTH release into the circulation provide a robust mechanism for maintaining a constant level of ionized calcium in blood [21]. Secreted intact PTH is extensively metabolized by liver and kidney and disappears from the circulation with a half-life less than 5 minutes. Therefore, the blood level of PTH is essentially determined by the activity of the parathyroid glands.

In addition to calcium and vitamin D as major regulators of PTH synthesis and secretion, other factors may affect serum PTH levels. For incidence, PTH secretion is modulated by the activity of the autonomous nervous system [22], [23]. Recently, hyperparathyroidism has been identified as a novel feature of primary aldosteronism [24]. Expression of the angiotensin II type I receptor and the mineralocorticoid receptor has been identified in normal parathyroid glands and found to be increased in adenomatous parathyroid glands [25]. These findings are consistent with the observation that in the general population, a high aldosterone-to-renin ratio was associated with high serum PTH concentrations [26].

Perchlorate, nitrate, and thiocyanate are well-known inhibitors of sodium-iodide symporter. Concomitant exposure to perchlorate, thiocyanate, and low iodine markedly reduced thyroxine production compared to exposure to each factor alone [27]. However, the effects on serum PTH levels have not been examined. Gallium nitrate has been used to treat intractable hypercalcemia. Gallium nitrate may reduce PTH release by stabilization of the plasma membrane rather than by interference with CaSR [28]. Although Ga^3+^ at 200 µM inhibited PTH release whereas 600 µM NO_3_^−^ had no effect [29], it could not completely exclude the possibility that nitrate may directly modulate PTH secretion. Smoking is a potential confounder of our results. In an animal model, nicotine reduced activity of parathyroid chief cells [30]. Nonetheless, there are inconsistent results with regard to the effects of smoking on PTH levels. Based on the NHANES data, smokers had lower PTH levels [15]. In another study, serum 25-hydroxyvitamin D levels and calcium absorption was lower in both light and heavy smokers, whereas PTH levels were higher in heavy smokers [31]. In patients with primary hyperparathyroidism, smoking was associated with lower PTH and higher phosphate levels [32]. Conversely, in dialysis patients with secondary hyperparathyroidism, heavy smoking was independently associated with high PTH levels [33]. In the present study, after adjusting for smoking status, the association between PTH level and urinary perchlorate, nitrate, and thiocyanate remains significant. The mechanisms of this intriguing association have to be elucidated.

We had several unexpected findings. For example, exposure to different anions was not associated with PTH to the same extent. It was found that the relative potency of perchlorate to inhibit iodide uptake was 15 and 240 times that of thiocyanate and nitrate [1]. However, we could not ascertain the mechanism(s) underlying the associations; therefore, it is impossible to compare directly. In addition, the inverse relation between perchlorate and PTH was observed mainly in women. Our previous study suggested that different target organ susceptibility to hyperparathyroidism may exist in different genders [34]. On the other hand, gender difference may have some impacts on PTH levels. In this study, males were found to have higher perchlorate, nitrate, and thiocyanate levels. It may represent a potential link to the fact that primary [35] and secondary [36] hyperparathyroidism occurs more frequently in women. Nonetheless, more studies are needed, particularly with respect to interactions involving demographic, lifestyle, dietary, and season factors [37].

Serum PTH levels may independently associate with mortality [38]. Recently, we demonstrated that PTH levels are associated with several inflammatory markers [39]. Prolonged elevation of PTH levels may result in bone loss, fractures, cardiovascular disease, and increased mortality [13]. Radiation and lithium therapy are predisposing factors in only a minority of sporadic primary hyperparathyroidism. For most patients, the etiology is unknown [40]. Although these anions from environmental and dietary sources negatively regulate PTH levels and are unlikely to account for the development of hyperparathyroidism, our results unveil the complex interaction between PTH regulation and other unknown factors.

There were several limitations to the current study. This study is cross-sectional in nature, therefore making it impossible to draw cause-and-effect inferences in the observed associations. Second, the association observed might not be related to the inhibitory effects on sodium-iodide symporter. It is uncertain whether these three anions influence serum PTH levels through the same pathophysiology. Third, our analysis is limited to the use of single spot urine samples to assess exposure, although previous reports indicated fair temporal reliability in the spot urine concentrations of the three anions [41].

## Conclusions

Using the NHANES data from the U.S. adult population, we found that a higher urinary concentration of perchlorate, nitrate, and thiocyanate is associated with lower serum PTH levels. Although the pathophysiological background of the association is as yet unclear, our observation may disclose novel regulatory controls with high clinical relevance. Future studies are needed to confirm or disprove our findings.
